# When words are missing: A personal reflection on accompaniment at the end of life

**DOI:** 10.1017/S1478951526101928

**Published:** 2026-02-24

**Authors:** Najlaa El Gouar

**Affiliations:** Higher Institute of Health Sciences (ISSS), LIDEALL Laboratory, Hassan First University, Settat, Morocco

I did not encounter palliative care as a clinician or a researcher. I encountered it as a daughter.

My mother was diagnosed with cancer in 2018. What followed were years marked by treatments, moments of hope, physical decline, and repeated encounters with the healthcare system. In early 2021, the disease progressed irreversibly. The final 3 months of her life were unmistakably different: persistent vomiting, profound fatigue, recurrent hospitalizations, and the gradual disappearance of therapeutic options. It was only then that the term *palliative care* was explicitly used. What remained was no longer cure, but presence.

This essay is not intended as a clinical case study. Nor does it seek to evaluate medical decisions. It is a reflective account of accompaniment, written from the position of a daughter navigating the final phase of her mother’s life, often without guidance, preparation, or language to understand what was unfolding. Through this reflection, I aim to illuminate the communication gaps, ethical tensions, and invisible burdens experienced by relatives in palliative care, and to question how accompaniment might be better supported.

## Learning a language that was never taught

Throughout my mother’s illness, communication with healthcare professionals was frequent, efficient, and technically dense. Consultations were often brief, focused on biological indicators and treatment protocols. Yet the language used remained largely inaccessible. Medical terms were rarely reformulated, and little space was provided to ensure understanding.

Gradually, my role shifted. I became an interpreter, attempting to translate diagnoses, procedures, and prognostic implications for my mother, and often for other patients encountered in waiting rooms and hospital corridors. This informal mediation was never formally assigned; it emerged from necessity.

What struck me most was not the absence of information but the assumption that families would adapt to medical discourse rather than the inverse. In palliative contexts, where emotional vulnerability is heightened and cognitive resources are strained, this asymmetry deepens confusion and anxiety. Communication, rather than alleviating suffering, risks becoming an additional burden.

Scholars in health communication have long emphasized that understanding is not guaranteed by information alone but requires dialogue, reformulation, and attentiveness to patients’ and families’ capacities (Street et al. 2009).

## Between hope and the delayed naming of palliative care

For much of the illness trajectory, palliative care was not named. Treatments continued, examinations were repeated, and silence surrounded the limits of what medicine could offer. In this silence, hope persisted, not necessarily because it was promised but because it was not explicitly challenged.

When palliative care was finally articulated, it was experienced as a rupture rather than a transition. Retrospectively, I wonder how different the final months might have been had this shift been introduced earlier, progressively, and with words that allowed emotional preparation.

Hope does not disappear when palliative care begins; it changes form. It may become hope for comfort, for presence, for meaningful connection. Without communication, however, families remain suspended between anticipation and denial, unsure how to accompany someone who is dying while still receiving care. Honest and compassionate conversations, although difficult, offer structure rather than despair (Bernacki and Block 2014).

## The invisible labor of being there

As curative options faded, presence became the central task. I accompanied my mother to appointments, remained at her bedside during long nights, managed practical details, and absorbed her fears when words failed her. This labor, emotional, relational, logistical, was constant.

Yet it remained largely invisible.

No framework existed to acknowledge or support this role. No one asked how I was coping, or whether I understood what was happening. I was expected to be available, resilient, and silent. Over time, this silence ceased to be a sign of strength and became a form of isolation.

The literature consistently documents the psychosocial burden carried by informal caregivers, particularly in palliative contexts, where emotional exhaustion and distress are common (Hudson et al. 2011). Yet recognition of this burden rarely translates into concrete support within care systems.

## Pain, lucidity, and ethical tension

One of the most challenging moments of accompaniment emerged around pain management. Strong medications alleviated physical suffering but altered my mother’s consciousness. Hallucinations appeared. Her sense of identity seemed fragmented.

At a certain point, she made a decision: she chose to reduce or discontinue some treatments, preferring pain to confusion. She wanted to remain lucid, to recognize us, to speak, to remain herself.

This choice unsettled us deeply. Witnessing pain when relief appeared technically available felt unbearable. What was missing was not consent but explanation. No one helped us understand that this was an ethical decision, rooted in autonomy, values, and dignity.

Without communication, such decisions risk being experienced by relatives as abandonment or failure, rather than as expressions of agency. Ethical clarity requires dialogue, not only between clinicians and patients but also with families who must live with the consequences of these choices.

## When words are missing

As death approached, something unexpected occurred: I lost the ability to speak.

I sat beside my mother knowing that time was limited. I knew what I felt. I knew what I wanted to say. And yet, no words came. Not because of indifference but because I had never been prepared for this moment.

This silence remains one of my deepest regrets, not a regret of absence but of unspoken presence.

Only later did I understand that accompaniment is not intuitive. It requires models, permission, and language. Without them, even love may fall silent. Psychological literature has long described this paralysis as a common response to confronting finitude, where emotion overwhelms expression (Kübler-Ross 1969).

### Concluding reflection

This reflection suggests that the suffering experienced by relatives in palliative care is not limited to grief. It is amplified by communication failures, lack of recognition, and absence of educational support.

Palliative care attends carefully to bodies. It must also attend to relationships.

If relatives are expected to accompany, they must be prepared, not to cure or to save but to be present with understanding, clarity, and compassion. Creating accessible educational spaces for families is not ancillary to care; it is part of care itself.

My hope is that this reflection contributes, modestly, to making accompaniment less lonely, less silent, and more humane.
